# Decelerated DNA methylation age predicts poor prognosis of breast cancer

**DOI:** 10.1186/s12885-018-4884-6

**Published:** 2018-10-17

**Authors:** Jun-Ting Ren, Mei-Xia Wang, Yi Su, Lu-Ying Tang, Ze-Fang Ren

**Affiliations:** 1 The School of Public Health, Sun Yat-sen University, 74 Zhongshan 2nd Road, Guangzhou, 510080 People’s Republic of China; 2 Department of Medicine and Therapeutics, Prince of Wales Hospital, The Chinese University of Hong Kong, Shatin, Hong Kong, China; 3 The Third Affiliated Hospital, Sun Yat-sen University, Guangzhou, China; 4 Mailman School of Public Health, Columbia University, New York, USA

**Keywords:** DNA methylation age, Breast cancer, Prognosis

## Abstract

**Background:**

DNA methylation (DNAm) age was found to be an indicator for all-cause mortality, cancer incidence, and longevity, but no study has involved in the associations of DNAm age with the prognosis of breast cancer.

**Methods:**

We retrieved information of 1076 breast cancer patients from Genomic Data Commons (GDC) data portal on March 30, 2017, including breast cancer DNAm profiling, demographic features, clinicopathological parameters, recurrence, and all-cause fatality. Horvath’s method was applied to calculate the DNAm age. Cox proportional hazards regression models were used to test the associations between DNAm age of the cancerous tissues and the prognosis (recurrence of breast cancer and all-cause fatality) with or without adjusting for chronological age and clinicopathological parameters.

**Results:**

The DNAm age was markedly decelerated in the patients who were premenopausal, ER or PR negative, HER2-enriched or basal-like than their counterparts. In the first five-year follow-up dataset for survival, every ten-year increase in DNAm age was associated with a 15% decrease in fatality; subjects with DNAm age in the second (HR: 0.52; 95%CI: 0.29–0.92), the third (HR: 0.49; 95%CI: 0.27–0.87) and the fourth quartile (HR: 0.38; 95%CI: 0.20–0.72) had significant longer survival time than those in the first quartile. In the first five-year follow-up dataset for recurrence, every ten-year increase in DNAm age was associated with a 14% decrease of the recurrence; in the categorical analysis, a clear dose-response was shown (*P* for trend =0.02) and the fourth quartile was associated with a longer recurrence free survival (HR: 0.32; 95%CI: 0.14–0.74). In the full follow-up dataset, similar results were obtained.

**Conclusions:**

DNAm age of breast cancer tissue, which associated with menopausal status and pathological features, was a strong independent predictor of the prognosis. It was suggested that the prognosis of breast cancer was related to intrinsic biological changes and specific molecular targets for treatment of breast cancer may be implicit.

## Background

Ageing presents numerous progressive changes in molecular, cellular, tissular and organismal functions, which ultimately drives various diseases and limits lifespan [1]. Consequently, age has been confirmed to be the strongest demographic risk factor for most common chronic human diseases, including cancers [2]. Ageing indicates accumulation of somatic mutations as well as aberrant epigenetic changes (epimutations) [3, 4]. Based on DNA methylation data, an age estimator (referred to as DNAm age) has been developed to accurately estimate chronological age across multiple normal tissues [5, 6]. An increasing body of literatures reported that the DNAm age was able to capture the aspects of the biological age of the underlying normal tissue and predict the susceptibility to various health outcomes. For example, the DNAm age of blood was predictive of all-cause mortality [7–11], cancer incidence [12–17], and longevity [10].

For malignant tumor tissues, however, the DNAm age was not able to estimate the chronological age of the host [6]. This may be because DNAm pattern in the clones of cancer origination is different from that of normal tissue and it only presents the state of ageing in the tumor cells [18]. It was exhibited that stem cells had the lowest DNAm age and this age increased when they differentiated into more mature cells [6]. Moreover, the cancer new clones develop with a wide variation, which consequently induces huge inter- and intra- heterogeneity in cancer tissues, including both the genomics and epigenomics [19]. Therefore, we speculate that the DNAm age of cancer cells may present the capacity to differentiate into malignant clones and can predict the outcome of the disease. Till now, only one study have involved in the associations of DNAm age in malignant diseases with the prognosis, while breast cancer was not included [18]. The role of DNAm age in tumor tissues in predicting the prognosis of cancer patients is far from being confirmed.

In the present study, we focused on breast cancer and comprehensively analyzed whether the DNAm age in tumor tissues was associated with the prognosis when taking the chronological age and the clinicopathological features into account, using the datasets from the Cancer Genome Atlas (TCGA) data portal.

## Methods

### Datasets

We retrieved all available breast cancer DNAm profiles on Infinium Human Methylation 450 Bead Chip or Human Methylation 27 Bead Chip (Illumina Inc.) from Genomic Data Commons (GDC) data portal (https://portal.gdc.cancer.gov/) with TCGA datasets using the R/Bioconductor TCGAbiolinks package [20] (https://www.bioconductor.org/). Corresponding demographic characteristics (gender, chronological age, menopausal status, and race), clinicopathological parameters (tumor stage and subtypes), follow up data (recurrence, all-cause fatality) were also downloaded from GDC on March 30, 2017. Thus, the present study dataset contains 1085 breast cancer DNAm profiles for 1076 female patients (9 subjects had double profiles which were averaged). Other 122 DNAm profiles for adjacent normal breast tissues were also included in the dataset to demonstrate the accuracy of the estimation method on chronological age. Only 889of these female breast cancer patients had recurrence free survival information which was obtained from UCSC Xena (http://xena.ucsc.edu/).

### DNAm age calculation

We applied Horvath’s method to calculate the DNAm age [6], which is currently the most robust predictor of chronological age [21]. Briefly, 353 dinucleotide markers were selected from 21,369 CpG probes on the Illumina 27 K and 450 K platforms with a penalized regression model in a large sample (*n* = 8000), including 51 healthy tissues and cell types and covering the entire adult life span. These markers were weighted to estimate the DNAm age (in units of years). It shows high age correlations (*r* = 0.96) and small mean deviation from calendar age (3.6 years) in its validation cohort. Mathematical details and software tutorials for DNAm age calculation can be found in the Additional files of Horvath [6]. An online age calculator (https://dnamage.genetics.ucla.edu) is available, by which the DNAm ages for the adjacent normal tissues and the cancerous tissues from the breast cancer patients in the dataset were obtained.

### Statistics

Scatter plots were generated to illustrate the relationship between chronological age and DNAm age in the adjacent normal tissues and cancerous tissues. Pearson correlation coefficient (r) between chronological and DNAm ages were computed accordingly. Cox proportional hazards regression models were used to test the associations between DNAm age of the cancerous tissues and the prognosis (recurrence of breast cancer and all-cause fatality). Hazard Ratios (HRs) and corresponding 95% confidence intervals (CIs) were calculated. Three models were applied: 1) no adjustment, 2) adjusted only for chronological age (continuous), and 3) further adjusted for race, clinical stage, menopause status, estrogen receptor (ER), human epidermal growth factor receptor 2 (HER2), and PAM50 subtype. DNAm age was regarded as either a linear function expressed by per ten-year increase or category of quartile.

Four endpoints were applied to present the prognosis: 1) overall survival (full follow-up), 2) five-year survival (the first five-year follow-up), 3) overall recurrence free survival (full follow-up), and 4) five-year recurrence free survival (the first five-year follow-up). Five-year survival and recurrence free survival were generated from the original dataset by censoring patients who died after five-year follow-up and limiting survival time to 5 years for patients who survived for more than 5 years.

Stratified analyses for the associations were performed by race, menopausal status and pathological characteristics of HER2, ER, PAM50 subtype, and clinical stage. The interactions between DNAm age and stratified variables were evaluated by adding an interaction term in the Cox model, which was tested by Wald test. All statistical tests were two-tailed with *P* < 0.05 considered to be significant. Statistical analyses were conducted using R software version 3.3.2 (https://www.r-project.org/).

## Results

### Relationship between chronological age and DNAm age

**Fig. 1:**
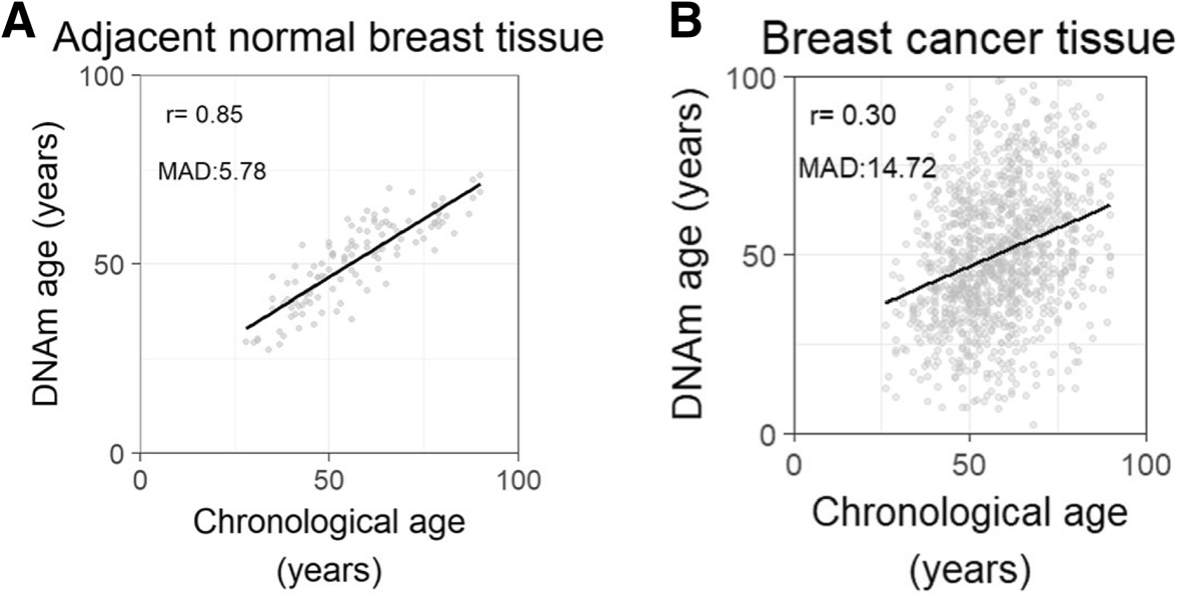
Correlations between DNAm age and chronological age. **a** DNAm age of 122 adjacent normal breast tissues from breast cancer patients can predict chronological age with decent accuracy. The median absolute deviation (MAD) and range of the difference between DNAm age and chronological age were 5.78 years and − 24.94 to 12.02 years, respectively. **b** DNAm age of 1097 breast cancers was poorly correlated with patients’ chronological age. The MAD and range of the difference between DNAm age and chronological age were 14.72 years and − 67.35 to 91.38 years, respectively

As shown in Fig. 1, the Pearson correlation coefficients between DNAm age and chronological age were 0.85 (*p* < 0.01) for breast normal tissues and 0.30 (*p* < 0.01) for breast cancerous tissues. The median absolute deviations (ranges of the difference between DNAm age and chronological age) were 5.78 (− 24.94 to 12.02) years and 14.72 (− 67.35 to 91.38) years for normal tissues and cancerous tissues, respectively.

### Characteristics and the relationships with DNAm age in breast cancer tissues

The demographic and clinicopathological characteristics for 1076 female breast cancer patients were shown in Table 1. The majority of the patients were over 40 years old. Chronological age, in a way of categorical variable, was positively associated with DNAm age. The African American patients had a significant lower DNAm age than the whites or others. The DNAm age was markedly decelerated among the patients who were premenopausal, ER or PR negative, HER2-enriched or basal-like than their corresponding counterparts.

**Table 1:** Demographic and clinicopathological characteristics and the associations with DNA methylation age for 1076 female breast cancer patients (N, %)

Characteristics	Total	DNAm age (quartile)^b^				*P* value ^a^
		First	Second	Third	Fourth	
Age (years)						
< 40	96 (8.9)	52 (19.3)	20 (7.4)	13 (4.8)	11 (4.1)	**< 0.01**
40–60	496 (46.1)	145 (53.9)	148 (55.0)	115 (42.8)	88 (32.7)	
> 60	484 (45.0)	72 (26.8)	101 (37.5)	141 (52.4)	170 (63.2)	
Race						
African American	178 (16.5)	72 (26.8)	40 (14.9)	35 (13.0)	31 (11.5)	**< 0.01**
White or other	898 (83.5)	197 (73.2)	229 (85.1)	234 (87.0)	238 (88.5)	
Menopausal status						
Premenopausal	229 (21.3)	80 (29.7)	57 (21.2)	54 (20.1)	38 (14.1)	**< 0.01**
Post-menopausal	736 (68.4)	153 (56.9)	185 (68.8)	187 (69.5)	211 (78.4)	
Unknown	111 (10.3)	36 (13.4)	27 (10.0)	28 (10.4)	20 (7.4)	
ER						
Negative	178 (16.6)	99 (36.8)	53 (19.7)	20 (7.4)	6 (2.2)	**< 0.01**
Positive	588 (54.6)	95 (35.3)	140 (52.0)	161 (59.9)	192 (71.4)	
Unknown	310 (28.8)	75 (27.9)	76 (28.3)	88 (32.7)	71 (26.4)	
PR						
Negative	251 (23.3)	122 (45.4)	67 (24.9)	36 (13.4)	26 (9.7)	**< 0.01**
Positive	512 (47.6)	70 (26.0)	126 (46.8)	145 (53.9)	171 (63.6)	
Unknown	313 (29.1)	77 (28.6)	76 (28.3)	88 (32.7)	72 (26.7)	
HER2						
Negative	644 (59.9)	156 (58.0)	162 (60.2)	155 (57.6)	171 (63.5)	0.33
Equivocal/positive	118 (11.0)	37 (13.7)	31 (11.5)	24 (9.0)	26 (9.7)	
Unknown	314 (29.1)	76 (28.3)	76 (28.3)	90 (33.4)	72 (26.8)	
PAM50						
Luminal A	229 (21.3)	25 (9.3)	51 (19.0)	71 (26.4)	82 (30.5)	**< 0.01**
Luminal B	122 (11.3)	22 (8.2)	25 (9.3)	28 (10.4)	47 (17.5)	
HER2-enriched & Basal-like	155 (14.4)	84 (31.2)	41 (15.2)	22 (8.2)	8 (3.0)	
Unknown	570 (53.0)	138 (51.3)	152 (56.5)	148 (55.0)	132 (49.0)	
Clinical stage						
I/II	568 (52.8)	136 (50.6)	149 (55.4)	136 (50.6)	147 (54.6)	0.26
III/IV	187 (17.4)	56 (20.8)	38 (14.1)	44 (16.3)	49 (18.2)	
Unknown	321 (29.8)	77 (28.6)	82 (30.5)	89 (33.1)	73 (27.1)	

^a^ Pearson chi-squared test. Unknown data was not accounted in the test

^b^Patients were divided into quartiles according to DNAm age. First quartile: 2.2–37.6; Second quartile: 37.7–49.8; Third quartile: 49.9–64.9; Fourth quartile: 65–157

Significant ones are in boldface

### Associations between DNAm age and prognosis

During the full follow-up period, 151 all-cause deaths were recorded in all patients and 96 cases recurred in 889 patients with recurrence data. During the first five-year follow-up period, 98 and 79 patients were recorded for all-cause deaths and recurrence, respectively.

When survival as an outcome, older DNAm age was associated with longer survival, and this association was more evident in the first five-year follow-up dataset, in which every ten-year increase in DNAm age was associated with a 15% decrease in fatality in the full adjustment model (HR: 0.85; 95%CI: 0.76–0.96) (Table 2). Compared with the first quartile, the second (HR: 0.52; CI: 0.29–0.92), the third (HR: 0.49; 95%CI: 0.27–0.87), and the fourth quartile (HR: 0.38; 95%CI: 0.20–0.72) were all associated with a longer survival in the first five-year follow-up dataset, and the *P* value for trend was significant (*P* = 0.004). In addition, compared with the first quartile, the combined three upper quartiles were also associated with a longer survival (HR: 0.47; 95%CI: 0.29–0.76). For all the endpoints, the associations between DNAm age and breast cancer prognosis were stronger after adjusted for chronological age.

**Table 2:** Association of overall and five-year survival with DNA methylation age

DNAm Age	N	Overall survival			Five-year survival		
		HR (95% CI) ^a^	HR (95% CI) ^b^	HR (95% CI) ^c^	HR (95% CI) ^a^	HR (95% CI) ^b^	HR (95% CI) ^c^
Continuous							
(Per ten year)	1076	1.01 (0.94–1.08)	0.92 (0.85–1.00)	0.94 (0.86–1.02)	0.92 (0.84–1.01)	**0.84 (0.75–0.93)**	**0.85 (0.76–0.96)**
Categorical (quartile) ^d^							
First	269	1.00 (Ref)	1.00 (Ref)	1.00 (Ref)	1.00 (Ref)	1.00 (Ref)	1.00 (Ref)
Second	269	0.80 (0.51–1.26)	0.66 (0.42–1.05)	0.64 (0.39–1.04)	0.59 (0.34–1.03)	**0.49 (0.28–0.85)**	**0.52 (0.29–0.92)**
Third	269	0.79 (0.51–1.24)	**0.56 (0.35–0.89)**	**0.56 (0.34–0.93)**	0.66 (0.39–1.12)	**0.46 (0.27–0.78)**	**0.49 (0.27–0.87)**
Fourth	269	1.00 (0.66–1.53)	**0.56 (0.35–0.90)**	0.60 (0.36–1.04)	0.60 (0.34–1.03)	**0.33 (0.18–0.59)**	**0.38 (0.20–0.72)**
P for trend		0.939	**0.014**	0.065	0.075	**0.001**	**0.004**
Categorical (First quartile and second-fourth quartile)							
First	269	1.00 (Ref)	1.00 (Ref)	1.00 (Ref)	1.00 (Ref)	1.00 (Ref)	1.00 (Ref)
Second-Fourth	807	0.86 (0.61–1.22)	**0.59 (0.41–0.86)**	**0.60 (0.40–0.91)**	**0.61 (0.41–0.93)**	**0.42 (0.27–0.65)**	**0.47 (0.29–0.76)**

^a^No adjustment

^b^Adjusted only for chronological age (continuous)

^c^Adjusted for chronological age (continuous), race, clinical stage, menopause status, ER status, HER2 status and PAM50 subtype

^d^Patients were divided into quartiles according to DNAm age. First quartile: 2.2–37.6; Second quartile: 37.7–49.8; Third quartile: 49.9–64.9; Fourth quartile: 65–157

Significant ones are in boldface

When recurrence free survival as an outcome, it was similarly shown that higher DNAm age was associated with a longer recurrence-free survival (Table 3). Every ten-year increase in DNAm age was significantly associated with a 14% decrease of the recurrence for both datasets of full and five-year follow-up in the full adjustment model. In the categorical analysis, a significant dose-response relationship was shown (*P* for trend < 0.05) and the fourth quartile was associated with a longer recurrence-free survival [HR (95%CI): 0.39 (0.19–0.80) and 0.32; 0.14–0.74 for full follow-up and five-year follow-up, respectively)], although the combined three upper quartiles were not significantly associated with recurrence-free survival when compared with the first quartile.

**Table 3:** Association of recurrence free survivals with DNA methylation age

DNAm Age	N	Overall recurrence free survival			Five-year recurrence free survival		
		HR (95% CI) ^a^	HR (95% CI) ^b^	HR (95% CI) ^c^	HR (95% CI) ^a^	HR (95% CI) ^b^	HR (95% CI) ^c^
Continuous							
(Per ten year)	889	**0.89 (0.81–0.98)**	**0.86 (0.77–0.96)**	**0.86 (0.76–0.97)**	**0.87 (0.78–0.98)**	**0.84 (0.75–0.95)**	**0.86 (0.75–0.98)**
Categorical (quartile) ^d^							
First	215	1.00 (Ref)	1.00 (Ref)	1.00 (Ref)	1.00 (Ref)	1.00 (Ref)	1.00 (Ref)
Second	230	0.82 (0.48–1.39)	0.76 (0.44–1.31)	0.77 (0.43–1.36)	0.77 (0.43–1.36)	0.70 (0.39–1.25)	0.71 (0.39–1.31)
Third	227	0.82 (0.49–1.37)	0.72 (0.42–1.23)	0.70 (0.39–1.29)	0.76 (0.43–1.36)	0.65 (0.36–1.17)	0.68 (0.36–1.32)
Fourth	217	**0.48 (0.25–0.90)**	**0.40 (0.21–0.78)**	**0.39 (0.19–0.80)**	**0.39 (0.19–0.80)**	**0.31 (0.15–0.66)**	**0.32 (0.14–0.74)**
P for trend		**0.031**	**0.009**	**0.014**	**0.014**	**0.003**	**0.020**
Categorical (First quartile and second-fourth quartile)							
First	215	1.00 (Ref)	1.00 (Ref)	1.00 (Ref)	1.00 (Ref)	1.00 (Ref)	1.00 (Ref)
Second-Fourth	674	0.71 (0.46–1.08)	**0.64 (0.41–1.00)**	0.66 (0.39–1.09)	0.64 (0.40–1.02)	**0.57 (0.35–0.93)**	0.62 (0.35–1.07)

^a^No adjustment

^b^Adjusted only for chronological age (continuous)

^c^Adjusted for chronological age (continuous), race, clinical stage, Menopause Status, ER status, HER2 status and PAM50 subtype

^d^Patients were divided into quartiles according to DNAm age. First quartile: 2.2–37.6; Second quartile: 37.7–49.8; Third quartile: 49.9–64.9; Fourth quartile: 65–157

Significant ones are in boldface

Stratified analyses were further performed to assess whether the associations between the DNAm age and the prognosis of breast cancer were modified by clinical-pathological characteristics and menopausal status (Table 4). Although the interactions did not reach the level of statistical significance, the subgroups showed considerable differences in HR estimates when stratified by menopause status. The HR and 95% CI (three upper combined quartiles vs. first quartile DNAm age) were 0.40 (0.24–0.69) in post-menopausal patients and 0.87 (0.30–2.58) in pre-menopausal patients for overall survival, and the HR and 95% CI were 0.58 (0.29–1.16) and 1.16 (0.42–3.21) for recurrence-free survival, respectively. A similar result was shown for HER2 status; the association of higher DNAm age with a better prognosis was stronger in HER2 positive than negative patients. When stratified by PAM50 subtype, the overall survival was markedly worse in the patients with HER2-enriched or Basal-like breast cancer than those with Luminal A or B breast cancer (*P* for interaction = 0.016), while this phenomenon did not occur for recurrence-free survival.

**Table 4:** Associations of overall survival and recurrence free survivals with DNA methylation age stratified by menopause and clinicopathological features

Stratified variables	DNAm age (quartile)^a^	Survival		Recurrence	
		N	HR^b^ (95% CI)	N	HR^b^ (95% CI)
Menopause Status					
Pre	First	80	1.00 (ref)	72	1.00 (ref)
	Second-Fourth	149	0.87 (0.30–2.58)	133	1.16 (0.42–3.21)
Post	First	153	1.00 (ref)	117	1.00 (ref)
	Second-Fourth	583	**0.40 (0.24–0.69)**	486	0.58 (0.29–1.16)
P for interaction			0.3761		0.963
Clinical stage					
Stage I&II	First	136	1.00 (ref)	103	1.00 (ref)
	Second-Fourth	432	1.24 (0.62–2.49)	341	0.80 (0.32–1.96)
Stage III&IV	First	56	1.00 (ref)	39	1.00 (ref)
	Second-Fourth	131	**0.39 (0.17–0.91)**	97	1.07 (0.39–2.92)
P for interaction			0.159		0.665
ER status					
negative	First	99	1.00 (ref)	77	1.00 (ref)
	Second-Fourth	79	0.82 (0.31–2.15)	61	0.60 (0.19–1.84)
positive	First	95	1.00 (ref)	67	1.00 (ref)
	Second-Fourth	493	**0.54 (0.29–0.99)**	385	0.77 (0.33–1.78)
P for interaction			0.092		0.9972
HER2 status					
negative	First	156	1.00 (ref)	119	1.00 (ref)
	Second-Fourth	488	0.92 (0.51–1.64)	382	1.14 (0.57–2.31)
Equivocal /positive	First	37	1.00 (ref)	25	1.00 (ref)
	Second-Fourth	81	0.37 (0.09–1.58)	63	0.18 (0.03–1.21)
P for interaction			0.217		0.133
PAM50					
Luminal A	First	25	1.00 (ref)	13	1.00 (ref)
	Second-Fourth	204	0.72 (0.17–2.97)	149	0.59 (0.11–3.24)
Luminal B	First	22	1.00 (ref)	16	1.00 (ref)
	Second-Fourth	100	0.65 (0.21–2.06)	73	0.29 (0.06–1.46)
HER2-enriched&Basal-like	First	84	1.00 (ref)	61	1.00 (ref)
	Second-Fourth	71	1.91 (0.67–5.41)	48	0.66 (0.17–2.53)
P for interaction			**0.016**		0.966

^a^ Patients were divided into quartiles according to DNAm age. First quartile: 2.2–37.6; Second quartile: 37.7–49.8; Third quartile: 49.9–64.9; Fourth quartile: 65–157

^b^ Adjusted for chronological age (continuous), race, clinical stage, menopause Status, ER status, HER2 status and PAM50 subtype. When one of the confounders was the variable for stratifying, it was not adjusted in the model

Significant ones are in boldface

## Discussion

Although younger DNAm age of normal tissues was widely showed to be associated with better health outcomes in previous studies [7–17], the present study showed that younger DNAm age in the cancerous tissues of breast would predict a poorer prognosis. Since a higher DNAm age means that the individual is at an older age than chronological age, which is likely induced by harmful environmental exposures, unhealthy lifestyles, susceptible heredity, or stochastic events, it is reasonable for an accelerated DNAm age of health tissues to be connected with poorer health status. However, the situations might be different in cancerous tissues. As we know, carcinogenesis was an evolutionary process, driven by stepwise, somatic cell mutations with sequential, sub-clonal selection, forming the so-called cancer stem cells with potential to proliferation and propagation [22, 23]. Like DNAm age of stem cells which was low and increased with the propagation in nature, lower DNAm age of cancer cells might present more vicious tumor with a more potential to proliferate [6], which supports our present result of the association between younger DNAm age and poorer prognosis of breast cancer. In addition, this result was also consistent with the following two facts: lower DNAm age in cancer cells was associated with higher rates of genetic mutations, including P53 [6]; black breast cancer patients had a worse cancer-free interval than white patients, while the formers had a lower DNAm age than the later ones [24].

The associations between DNAm age and overall survival had ever been explored in several other tumors by Lin and Wagner and the associations were varied by the tumors derived organs [18]. The overall survival was more likely to be better in patients with esophageal carcinoma or glioblastoma multiforme if the DNAm age was older, which is in line with the result of present study, while a better prognosis with a younger DNAm age was showed in patients with thyroid carcinoma or renal clear cell carcinoma. There were no significant associations for cancers of lung, pancreas, skin, uterine, colon, bladder, et al. Based on these results, Lin and Wagner speculated that alterations of DNAm age could resemble a double edged sword [18]: on the one hand, the alterations may provide a barrier of proliferation for aging cells and prevent cancer initiation; on the other hand, they could also favor chromosomal changes that trigger other mutations, which might be the reason why increased DNAm age in different cancers had various effects on the prognosis. In the present study, it was also found that patients with different subtypes of breast cancer, such as Luminal A or B and HER2 enriched or Basal-like, had opposite associations between the DNAm age and the prognosis. Nevertheless, the comprehensive associations of DNAm age with various cancers or subtypes and the mechanisms are remained to be explored.

We further found that the association of higher DNAm age with a better prognosis might be stronger in post-menopausal than pre-menopausal patients, which was supported to some extent by the results in a recent published report in which accelerated DNAm age was found to be associated with breast cancer susceptibility only in postmenopausal but not pre-menopausal women [25]. It suggests that hormones may influence the associations between DNAm age and the initiation and development of breast cancer and the DNAm age may reflect the real biological age in a less-hormone condition. This is also supported by the facts that the DNAm age of female breast tissue is higher than that of their blood cells and the difference diminishes with increasing age [26]. It may also be explained by the roles of the age-associated compromised detoxification, DNA repair mechanisms and immune surveillance [27].

In the present analysis, we adjusted various factors and applied several outcomes, and the associations between DNAm age and breast cancer prognosis were consistent and quite strong. Chronological age seemed play a negative confounding role and the association between DNAm age and breast cancer prognosis was stronger when adjusted by chronological age, which can be explained by the facts that breast cancer prognosis was getting worse with the increase of chronological age [28], while DNAm age in tumors had a positive relationship with chronological age. However, the relationship between DNAm age in tumors and chronological age was weak and the negative effect on the association between DNAm age and breast cancer prognosis was not fundamental (as shown in Tables 2 and 3). As for the clinicopathological features, although the statuses of hormone receptors (ER and PR) and PAM50 subtype were associated with DNAm age, they only had a minor effect on the associations between DNAm age and breast cancer prognosis, indicating that the effect of DNAm age on breast cancer prognosis was not likely to mediate through the clinicopathological features.

We used four types of outcomes for breast cancer prognosis: overall survival, overall recurrence free survival, five-year survival, and five-year recurrence free survival, which have different clinical meanings. Overall survival means any survived patients including those who died of breast cancer as well as other diseases; the longer the time elapsed, the more patients died of other diseases. Therefore, the (five-year) recurrence free survivals might be better outcomes to estimate the prognosis specific to breast cancer, in which there was an obvious dose-response relationship between DNAm age and the outcomes (as shown in Table 3).

## Conclusion

In summary, the present study found that DNAm age of the tumor tissue, which associated with menopausal status and pathological features, was a strong independent predictor of breast cancer prognosis. These results suggested that the prognosis of breast cancer was related to intrinsic biological changes, and specific molecular targets for treatment of breast cancer may be implicit, particularly for that DNAm changes are of interest suggesting possible rejuvenation and health maintenance due to the reversibility [29]. The exact mechanisms and related genetic or environmental factors for the DNAm age remain to be explored.

## Abbreviations

CI: Confidence interval
DNAm: DNA methylation
ER: Estrogen receptor
GDC: Genomic Data Commons
HER2: Human epidermal growth factor receptor 2
HR: Hazards ratio
TCGA: The Cancer Genome Atlas
